# Estimating minute ventilation and air pollution inhaled dose using heart rate, breath frequency, age, sex and forced vital capacity: A pooled-data analysis

**DOI:** 10.1371/journal.pone.0218673

**Published:** 2019-07-09

**Authors:** Roby Greenwald, Matthew J. Hayat, Evi Dons, Luisa Giles, Rodrigo Villar, Djordje G. Jakovljevic, Nicholas Good

**Affiliations:** 1 Division of Environmental Health, School of Public Health, Georgia State University, Atlanta, Georgia, United States of America; 2 Division of Epidemiology and Biostatistics, School of Public Health, Georgia State University, Atlanta, Georgia, United States of America; 3 Flemish Institute for Technological Research (VITO), Mol, Belgium; 4 Centre for Environmental Sciences, Hasselt University, Diepenbeek, Belgium; 5 Sport Science Department, Douglas College, Vancouver, British Columbia, Canada; 6 Faculty of Kinesiology and Recreation Management, University of Manitoba, Winnipeg, Manitoba, Canada; 7 Institute of Cellular Medicine, Faculty of Medical Sciences, Newcastle University, Newcastle, United Kingdom; 8 Department of Environmental and Radiological Health Sciences, Colorado State University, Fort Collins, Colorado, United States of America; Univ Rennes, FRANCE

## Abstract

Air pollution inhaled dose is the product of pollutant concentration and minute ventilation (${\dot{V}}_{E}$). Previous studies have parameterized the relationship between ${\dot{V}}_{E}$ and variables such as heart rate (HR) and have observed substantial inter-subject variability. In this paper, we evaluate a method to estimate ${\dot{V}}_{E}$ with easy-to-measure variables in an analysis of pooled-data from eight independent studies. We compiled a large diverse data set that is balanced with respect to age, sex and fitness level. We used linear mixed models to estimate ${\dot{V}}_{E}$ with HR, breath frequency (f_B_), age, sex, height, and forced vital capacity (FVC) as predictors. FVC was estimated using the Global Lung Function Initiative method. We log-transformed the dependent and independent variables to produce a model in the form of a power function and assessed model performance using a ten-fold cross-validation procedure. The best performing model using HR as the only field-measured parameter was ${\dot{V}}_{E}$ = e^-9.59^HR^2.39^age^0.274^sex^-0.204^FVC^0.520^ with HR in beats per minute, age in years, sex is 1 for males and 2 for females, FVC in liters, and a median(IQR) cross-validated percent error of 0.664(45.4)%. The best performing model overall was ${\dot{V}}_{E}$ = e^-8.57^HR^1.72^f_B_^0.611^age^0.298^sex^-0.206^FVC^0.614^, where f_B_ is breaths per minute, and a median(IQR) percent error of 1.20(37.9)%. The performance of these models is substantially better than any previously-published model when evaluated using this large pooled-data set. We did not observe an independent effect of height on ${\dot{V}}_{E}$, nor an effect of race, though this may have been due to insufficient numbers of non-white participants. We did observe an effect of FVC such that these models over- or under-predict ${\dot{V}}_{E}$ in persons whose measured FVC was substantially lower or higher than estimated FVC, respectively. Although additional measurements are necessary to confirm this finding regarding FVC, we recommend using measured FVC when possible.

## Introduction

The public health consequences of ambient air pollution have been well-documented by more than three decades of epidemiologic, observational and clinical studies. The global burden of disease attributable to ambient air pollution is at a historical high and was estimated to be greater than 3 million deaths per year in 2010 [1]. Although air quality has improved significantly in recent decades in many parts of the developed world, ambient air pollution continues to present a formidable public health burden and is estimated to lead to over 200,000 premature deaths per year [2] in the United States. Efforts to better understand the causal relationships between environmental exposures and health effects are hampered by exposure misclassification, which can obscure the true association between exposure and disease and bias effect estimates [3–5]. Although much of the exposure misclassification in air pollution studies is spatial in nature, vast differences in ventilation rate between individuals also contribute to exposure misclassification.

Given that air pollution inhaled dose is a function of both pollutant concentration and the inhaled volume of air, it is important to accurately account for minute ventilation (${\dot{V}}_{E}$, the volume of air inhaled per minute) in order to reduce this misclassification and advance the science of air pollution exposure assessment. Although ${\dot{V}}_{E}$ is difficult or intrusive to measure in natural settings, in this paper, we describe a methodology for estimating ${\dot{V}}_{E}$ using data that is easily obtainable using wearable devices.

Several previous studies have estimated ${\dot{V}}_{E}$ from measurements of heart rate (HR) [6–9], breath frequency (f_B_) [10], power expenditure [11], or metabolic equivalents (METs) [12], and are succinctly summarized by Dons et al [13]. The typical model structure in these previous studies includes ${\dot{V}}_{E}$ as the dependent variable and HR or other physiological parameters as the predictors, often log-transformed. Since there is tremendous inter-subject variability in the relationship between ${\dot{V}}_{E}$ and HR, these models suffer from poor generalizability and typically have a wide range of percent error. A small-scale pilot study by the lead author of this paper collected data from fifteen adolescent athletes [14] and used the novel approach of using ${\dot{V}}_{E}$ normalized by forced vital capacity (${\dot{V}}_{E}/\mathrm{FVC}$) as the dependent variable. This approach effectively models the *fraction* of lung capacity an individual inhales per minute rather than the absolute volume of air and has the effect of reducing inter-subject variability in the relationship between ${\dot{V}}_{E}$ and physical activity. However, major limitations of this pilot study were small sample size and a non-representative study population. To address these concerns, we sought to explore a variety of methodologies in a much larger and more diverse data set by pooling data from several previously published studies.

## Methods

### Data collection

We performed a Pubmed database search with the terms “heart rate”, “breathing rate” and “minute ventilation” and narrowed the scope to papers with publication dates in the previous five years. This search returned 327 results, and upon closer examination, we identified 24 studies in which the abstract or main text indicated that all three parameters were measured in healthy humans with time resolution of one minute or less. These studies had a wide variety of scientific objectives, and only a few were related to air pollution exposure. In addition, we had previously identified six publications that were focused on estimating air pollution dose but did not meet our Pubmed database search criteria. We contacted by email the corresponding authors for all 30 identified papers and invited their participation in this analysis. Twelve authors responded agreeing to participate (in one case, the principal investigator had retired and the funding agency agreed on his behalf), two authors responded that the data was unavailable, and we received no response from the remaining 16 after two attempts. Of the twelve positive responses, ten investigators submitted data, of which eight data sets were usable for this analysis. Data from one study was unusable due to misaligned time stamps and another due to poor quality heart rate data (in neither case was this important for the original purpose of the respective study). The final eight participating studies produced a data set that includes 14,550 one-minute data points from 471 unique individuals in the age range of 4–80 years. The data set is balanced with respect to sex, includes individuals of a variety of different fitness levels from five different countries on three different continents, and is racially and ethnically diverse (though disproportionately white). In all cases, the data was deidentified, and a summary of subject characteristics is provided in Table 1. The participating studies are Greenwald et al. [14], Cozza et al. [8], Ramos et al. [9], Adams et al. [15], Giles et al. [16], Jakovljevic et al. [17], Villar et al. [18], and Good et al.[19]. All studies were approved by their respective Institutional Review Boards.

**Table 1:** Subject characterisitcs.

Age in years^a^	33(4–80)	
Height [cm]^b^	163±18	
Weight [kg]^b^	63±21	
BMI^a^	23.2(13.1–43.9)	
	Number of subjects	Number of data points
Race/ethnicity^c^		
Caucasian	373(79)	12306(83)
African-American	20(4.2)	534(3.6)
Hispanic	53(11)	1282(8.7)
Asian	25(5.8)	619(4.2)
Sex^b^		
Male	240(51)	8473(57)
Female	231(49)	6268(43)
Height [cm]^b^	163±18	
Weight [kg]^b^	63±21	
BMI^a^	23.2(13.1–43.9)	

^a^ Values are mean(range)

^b^ Values are mean±SD

^c^ Values are frequency(percentage)

An inherent strength of a pooled-data study is the greater level of generalizability that arises from analyzing data collected following diverse protocols using a variety of methodologies, instrumentation, and personnel. The data assembled in this paper include subjects at rest, sitting, standing, walking, running, cycling, and performing routine activities in an ambulatory setting. A summary of study protocols and data collection methodologies is provided in Table 2.

**Table 2:** Summary of study protocols.

	lung function	${\dot{V}}_{E}$	HR	f_B_	MET	motion	physical activity	number of subjects
Greenwald et al.	a	b	f	i	-	k	l	15
Cozza et al.	a	b	f	-	-	-	m	50
Ramos et al.	-	c	g	c	-	-	l	20
Adams et al.	-	b	f	b	-	-	l, n	212
Giles et al.	a	c	h	c	-	-	m	18
Jakovljevic et al.	-	d	f	d	j	-	l	79
Villar et al.	-	b	f	b	j	-	l	20
Good et al.	-	e	f	e	j	k	l, m, n	57

Abbreviations: ${\dot{V}}_{E}$−minute ventilation, HR–heart rate, f_B_−breath frequency, MET–metabolic equivalent, motion– 3-dimensional acceleration

- parameter was not measured

a spirometry

b vane respirometer

c pneumotachometer

d pitot tube flow sensor

e flat fan digital volume transducer

f R-R interval derived from electrocardiogram, electrodes worn on torso

g reflectance pulse oximetry, sensor worn on torso

h transmittance pulse oximetry, sensor worn on fingertip or ear

i chest expansion strap

j Metabolic Equivalent (MET), measured with wearable sensor

k accelerometer

l treadmill

m cycle ergometer

n field activities including walking, cycling, and specific tasks

### Model selection

We devoted considerable effort to exploring a wide variety of modeling approaches in order to identify the most appropriate and best performing predictive models. Given that the primary rationale for this study was to develop a practical model for assessing air pollution inhaled dose in field studies, we focused on modeling methodologies that are easily-implemented in a variety of applications and predictor variables that may be easily and inexpensively measured with high time-resolution. We therefore developed a list of potential predictors that included HR, f_B_, age, sex, height and weight as well as second order and/or interaction terms for these predictors. Due to their limited practicality in ambulatory settings, we did not include tidal volume (V_T_), metabolic equivalents (METs) or oxygen consumption (VO_2_) as predictors. In addition, given that the relationships of HR and ${\dot{V}}_{E}$ as well as f_B_ and ${\dot{V}}_{E}$ are non-linear [20] and have different response and relaxation times following stimuli [21], we examined the effect of log-transforming predictor variables and/or the independent variable as well as including HR and f_B_ lags (value 1, 2, 3, or 4 minutes previously) and factorials (current value multiplied by the value 1, 2, 3, and up to 4 minutes previously) as predictors.

A source of inter-subject variability in ${\dot{V}}_{E}$ is differences in lung volume, and we therefore explored four distinct modeling approaches to parameterize the effect of this variability. We ultimately rejected the first three of these approaches, but we will briefly describe them in order to justify our model selection. A common measure of functional lung volume is forced vital capacity (FVC), or the volume of air that can be exhaled with maximum effort, and another lung function parameter potentially useful for predicting ${\dot{V}}_{E}$ is forced expiratory volume in 1-second (FEV_1_). In persons with normal lung function, FEV_1_ is about 80% of FVC. In persons with obstructive airway disease such as asthma or COPD, FEV_1_ can be reduced relative to FVC, and during intense physical activity, this reduction in the ability to rapidly exhale may be relevant for the estimation of ${\dot{V}}_{E}$. FVC and FEV_1_ in healthy individuals are strongly correlated with height, and to a lesser extent, with age, sex, and race [22, 23]. Several previous well-powered studies [22, 23] have parameterized the influence of these variables on lung function and have developed algorithms for predicting FVC and FEV_1_. Our exploratory but ultimately rejected modeling approaches are labelled Approaches A-C (see Table A in the Supporting Information file titled S1 Text): Approach A uses ${\dot{V}}_{E}$ normalized by FVC as the dependent variable, Approach B uses ${\dot{V}}_{E}$ as the dependent variable and includes determinant factors of FVC as predictors, and Approach C uses the same as approach B but also includes FVC as a predictor of ${\dot{V}}_{E}$. As we describe below, the best-performing modeling approach is referred to as Approach D and uses log-transformed ${\dot{V}}_{E}$ as the dependent variable and log-transformed HR, f_B_, FVC, and subject-specific traits as predictor variables.

For Approaches A, C and D, we used the measured value of FVC or FEV_1_ in the subset of data for which it was available, and we also examined the entire dataset using predictions of FVC based on height, age, sex, and race or ethnicity according to the method of the Global Lung Function Initiative [23]. This method uses five racial or ethnic categories: Caucasians, African-Americans, North East Asians, South East Asians, and an Other category for all other ethnicities. We categorized white subjects from the United Kingdom, Portugal, Brazil, Canada, and the United States as Caucasian. All subjects of African ancestry were American and assigned to the African-American category. There were 25 American or Brazilian subjects listed as Asian; however, with no additional information regarding North or South Asian ancestry, we assigned these subjects to the Other category. In addition, there were 53 American subjects who self-identified as Hispanic, but again, with no additional knowledge of racial or national ancestry, these subjects were classified as Other. FVC or FEV_1_ predictions obtained using this method will not capture changes incurred by airway disease; however, the subjects enrolled in all studies were stated to be healthy.

### Statistical methods

We used general linear mixed models to reduce the inherent bias of within-subject repeated measures data [24]. All models were performed using the lme4 or nlme packages for R v3.2.2 (R Foundation for Statistical Computing). Presented results are from the lme4 package, while the nlme package was used to investigate covariance matrix structure. In particular, we examined the effect on model performance of using the variance components and first order autoregressive covariance matrix structures, and the best performing models used the variance components structure. We created a categorical variable called “study” that corresponds to each of the contributing studies. We included a random effect for subject and a random slope for both HR and f_B_ with subject. We additionally evaluated the effect of including a random effect for “study” to account for systematic differences between each study, although this random effect was not found to be important or improve model performance. We visually evaluated residual plots and did not observe evidence of heteroscedasticity. P-values were calculated for each predictor by using likelihood ratio tests to compare the full model with the predictor in question to the reduced model without. The level of significance was set *a priori* at 0.05.

### Cross validation

We performed a ten-fold cross validation procedure to assess model performance. Subjects were randomly divided into ten groups such that each group was comprised of a training set of 423 or 424 subjects and a validation set of 47 or 48. Parameter estimates were calculated based on the training sets, predictions were made for the validation sets, and then the predictions from all ten validation sets were assembled and compared with observations. The cross validated percent error was calculated as (predictions-observations)/observations·100%. We evaluated both model accuracy and precision by examining median percent error (favoring models with a smaller absolute value) and inter-quartile range (IQR, favoring models with a smaller spread in the distribution).

## Results

### Modeling approach

Parameter estimates and results for the best-performing models using approaches described above as A, B, or C (i.e. the dependent variable was not log-transformed, regardless of whether predictor variables were log-transformed) are included in S1 Text. Using these approaches, we observed substantial evidence of interaction between several predictor variables, namely HR with f_B_, and both HR and f_B_ with either FVC or the determinants of FVC (i.e. age, height, and sex). By this we mean that the p-values of these interaction terms were significant, and addition of these terms improved cross-validation predictive performance. In addition, we observed a significant effect of adding a second order term for HR (Table A in S1 Text). The interaction of FVC (or its determinants) with HR or f_B_ was reduced for Approach A, and as a consequence, it generally performed better than Approaches B or C. This can likely be explained by noting that the difference between Approach A and C is analogous to algebraically rearranging Eq 1 to produce Eq 2:

$${\dot{V}}_{E}/FVC=\beta_{0}+\beta_{1}HR+\beta_{2}f_{B}$$ (1)

$${\dot{V}}_{E}=\beta_{0}∙FVC+\beta_{1}HR∙FVC+\beta_{2}f_{B}∙FVC$$ (2)

Eq 3 expresses this as a hierarchically well-formulated model:

$${\dot{V}}_{E}=\beta_{0}^{\prime }+\beta_{1}^{\prime }HR+\beta_{2}^{\prime }f_{B}+\beta_{3}FVC+\beta_{4}HR*FVC+\beta_{5}f_{B}*FVC$$ (3)

In other words, Approach A moves the interaction of FVC (or its determinants) to the left-hand side of the model. Approach B is similar except that FVC is substituted with a function of age, height, and sex, leading to an even more complicated arrangement of interaction terms. The above equations are simplified in that HR, f_B_, and FVC are the only predictors shown, but the best performing models using these approaches also included a second order term for HR, interaction of HR with f_B_, age, height, and sex. An additional drawback to these approaches is related to the fact that the pooled dataset for this analysis includes a large number of data points from subjects at rest (approximately 10%). Approaches A, B, and C performed poorly for subjects at rest and occasionally produced negative predictions of ${\dot{V}}_{E}$ for subjects with HR of less than about 60 beats per minute. The minimum observed ${\dot{V}}_{E}$ for a subject at rest was 0.78·FVC, and we therefore substituted 0.78·FVC for any predicted ventilation value less than that for models using Approaches A, B, or C.

The difference between Approaches B and C could be characterized as a statistical power issue. By including predictors of FVC (height, age, sex, race), but not predicted FVC, Approach B essentially attempts to duplicate the FVC predictions of the GLI study, only with a smaller sample size and less statistical power. Approach C on the other hand leverages the larger sample size of the GLI study to produce better predictions of ${\dot{V}}_{E}$ than Approach B.

After observing the increasing model complexity and poor performance at rest, we evaluated Approach D, which uses a log-transformed dependent variable as well as log-transformed predictor variables. There are several notable advantages to using a model of this form: it eliminates the need for higher order terms for any predictor variable, it cannot produce a nonsensical negative prediction, and interaction terms between predictors are implicit. Eq 4 illustrates a simple model using only HR and f_B_ as predictors with the log-transformed interaction term between them explicitly included:

$$ln\left({\dot{V}}_{E}\right)=\beta_{0}+\beta_{1}ln(HR)+\beta_{2}ln(f_{B})+\beta_{3}ln(HR*f_{B})$$ (4)

This can be rearranged to give:

$$ln\left({\dot{V}}_{E}\right)=\beta_{0}+ln{(HR}^{\beta_{1}})+ln({f_{B}}^{\beta_{2}})+ln({HR}^{\beta_{3}}*{f_{B}}^{\beta_{3}})$$ (5)

$${\dot{V}}_{E}={exp[\beta }_{0}+ln{(HR}^{\beta_{1}})+ln({f_{B}}^{\beta_{2}})+ln({HR}^{\beta_{3}}*{f_{B}}^{\beta_{3}})]$$ (6)

$${\dot{V}}_{E}=e^{\beta_{0}}{HR}^{\beta_{1}}{f_{B}}^{\beta_{2}}{HR}^{\beta_{3}}{f_{B}}^{\beta_{3}}$$ (7)

Eq 8 shows the same model without an explicit interaction term:

$$ln\left({\dot{V}}_{E}\right)=\beta_{0}+\beta_{1}^{\prime }ln(HR)+\beta_{2}^{\prime }ln(f_{B})$$ (8)

This can be rearranged to give:

$${\dot{V}}_{E}=e^{\beta_{0}}{HR}^{\beta_{1}^{\prime }}{f_{B}}^{\beta_{2}^{\prime }}$$ (9)

Evaluation of the above models with and without an explicit interaction term shows that indeed $\beta_{1}^{\prime }=\beta_{1}+\beta_{3}$ and $\beta_{2}^{\prime }=\beta_{2}+\beta_{3}$ such that Eqs 7 and 9 are equivalent. This obviates the need for explicitly including interaction terms or higher order terms such as HR^2^. Although the best-performing model using Approach A is similar in cross-validated performance (when corrected for values less than 0.78·FVC) to the best model using Approach D, the Approach D models are much simpler and easier to evaluate, and therefore, all presented results are from Approach D.

### Best-performing models

**Fig 1:**
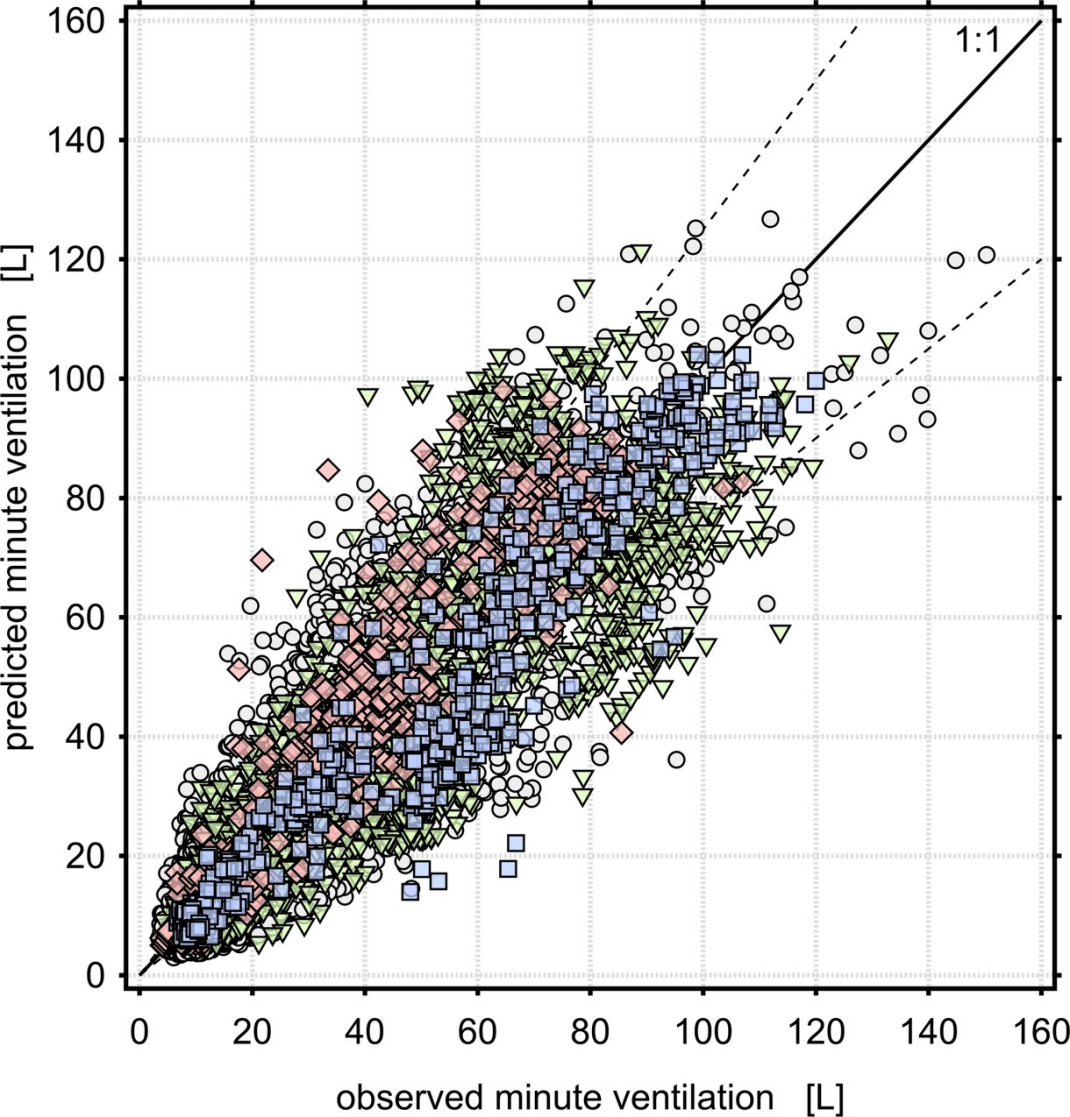
Cross validation results for Model D1: ${\dot{V}}_{E}$ = e^-9.59^HR^2.39^age^0.274^sex^-0.204^FVC^0.520^, where HR is beats per minute, age is in years, FVC is the GLI predicted value expressed in liters, and sex is 1 for males and 2 for females. The median(IQR) percent error from cross-validation for this model is -0.664(45.4)%. Circles are persons without an FVC measurement; triangles are persons with measured FVC = 85–115% of the predicted value; diamonds are persons with measured FVC < 85% predicted, and squares are persons with measured FVC > 115% predicted. Dashed lines are ±25% error.

**Fig 2:**
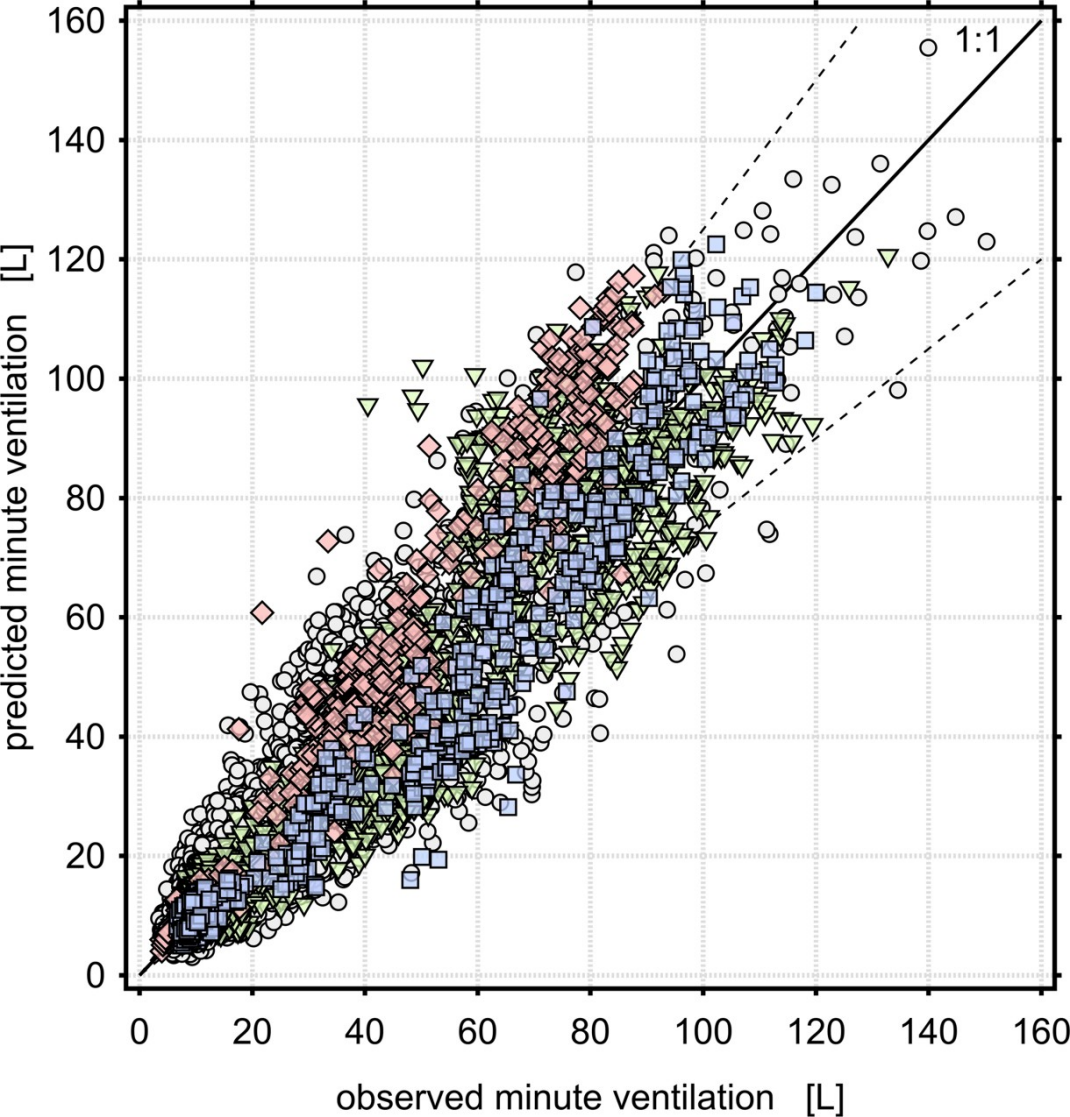
Cross validation results for Model D2: ${\dot{V}}_{E}$ = e^-8.57^HR^1.72^fB0.611age^0.298^sex^-0.206^FVC^0.614^, where HR is beats per minute, f_B_ is breaths per minute, age is in years, FVC is the GLI predicted value expressed in liters, and sex is 1 for males and 2 for females. The median(IQR) percent error from cross validation for this model is 1.20(37.9)%. Circles are persons without an FVC measurement; triangles are persons with measured FVC = 85–115% of the predicted value; diamonds are persons with measured FVC < 85% predicted, and squares are persons with measured FVC > 115% predicted. Dashed lines are ±25% error.

Given that HR is easier to measure in field studies than f_B_ and consumer- or medical-grade wearable devices for measuring HR have greatly proliferated in recent years, we separately evaluated models using HR as the only continuously-measured variable. The best-performing of these is labeled Model D1 in Table 3, and the cross-validation results are shown in Fig 1. Models including f_B_ as a predictor have noticeably improved predictive performance (in that the IQR of the cross-validation error is reduced). The best-performing of these is labeled Model D2 in Table 3, and the cross-validation results are shown in Fig 2. Due to the fact that one of the contributing studies did not measure f_B_, there were 471 subjects and 14550 data points available for estimating Model D1, but only 421 subjects and 13767 data points available for Model D2.

**Table 3:** Results of general linear mixed models using log-transformed ${\dot{V}}_{E}$ as the dependent variable, and all predictor variables are likewise log-transformed. These models yield a power function of the form ${\dot{V}}_{E}=e^{\beta_{0}}{HR}^{\beta_{1}}{f_{B}}^{\beta_{2}}{age}^{\beta_{3}}{sex}^{\beta_{4}}{FVC}^{\beta_{5}}$ where β_0_ is the model intercept.

	Model D1	Model D2
Intercept^a^	-9.59(-9.85,-9.32)	-8.57(-8.81,-8.33)
	p < 10^−6^	p < 10^−6^
HR [min^-1^]^a^	2.39(2.34,2.44)	1.72(1.66,1.78)
	p < 10^−6^	p < 10^−6^
f_B_ [min^-1^]^a^	-	0.611(0.560,0.661)
		p < 10^−6^
age [years]^a^	0.274(0.230,0.318)	0.298(0.262,0.335)
	p < 10^−6^	p < 10^−6^
sex [1 for males and 2 for females]^a^	-0.204(-0.287,-0.121)	-0.206(-0.277,-0.136)
	p < 10^−6^	p < 10^−6^
FVC [L]^a^	0.520(0.436,0.605)	0.614(0.546,0.681)
	p < 10^−6^	p < 10^−6^
percent error^b^	-0.664(45.4)	1.20(37.9)

^a^ The first row is the estimate(95% confidence intervals), and the second row is the p-value.

^b^ Percent error is the difference between predictions and observations from cross validation, and values are median(IQR).

## Discussion

### Effect of FVC

Only three of the eight contributors performed baseline lung function measurements [8, 14, 16, 25]. These included 83 unique subjects and 4,226 one-minute data points, and these subjects were disproportionately high-performing athletes. As a consequence, our models using measured baseline FVC as a predictor have substantially less statistical power than models using estimated FVC. On the other hand, given that both airway disease and genetic diversity can result in large differences between an individual’s predicted and actual FVC, FVC measurements have the advantage of capturing the effect of these differences on ${\dot{V}}_{E}$. The cross-validation results shown in Figs 1 and 2 use predicted FVC as a predictor variable, but data points are shape- and color-coded based on measured FVC. These results suggest that how well an individual’s predicted lung function agrees with measured lung function has an important influence on predictions of ${\dot{V}}_{E}$. Table 4 describes the results of Model D2 stratified by lung function status. ${\dot{V}}_{E}$ is substantially overestimated for persons with lower than normal lung capacity and underestimated for persons with higher than normal FVC. ${\dot{V}}_{E}$ is somewhat underestimated for persons with measured FVC close to the predicted volume, though to a lesser extent than persons with high FVC. Persons with unmeasured FVC are somewhat overestimated. These results are similar in other models including FVC as a predictor, but are exaggerated in models that do not include FVC. Given that ${\dot{V}}_{E}$ is increased during physical activity by increasing tidal volume as well as f_B_, and that tidal volume is related to FVC, the observation that ${\dot{V}}_{E}$ is overestimated for persons with low FVC is consistent with these persons having lower than normal tidal volume as well. Since tidal volume cannot be easily measured in ambulatory settings, our findings support the use of FVC measurements as an appropriate proxy to adjust for the effect of lung volume. Table 5 describes similar results as Table 4 except measured FVC is used to predict ${\dot{V}}_{E}$. Note that these results are still from Model D2 wherein parameter estimates are calculated based on predicted FVC. ${\dot{V}}_{E}$ predictions using measured FVC are substantially more accurate for persons with measured FVC differing from the predicted volume in either direction, and the distribution of error is more symmetrical. We therefore recommend that predictions of ${\dot{V}}_{E}$ using Models D1 or D2 be made using measurements of FVC if possible, particularly for persons with non-normal lung function.

**Table 4:** Effect of lung capacity on predictions of ${\dot{V}}_{E}$ using calculated FVC as a predictor according to Model D2. Model performance is shown for subjects with and without FVC measurements, and subjects with FVC measurements are further stratified into low, high, and normal FVC groups.

	percent error median(IQR)^a^	over (under)^b^	25% over (25% under)^c^	N^d^
all subjects	1.20(37.9)	52(48)	23(14)	13767
subjects with no FVC measurement	2.40(39.7)	53(47)	24(14)	10311
subjects with measured FVC 85–115% predicted	-4.00(32.1)	42(58)	18(16)	2288
subjects with measured FVC < 85% predicted	15.4(26.9)	78(22)	32(2)	596
subjects with measured FVC > 115% predicted	-9.42(24.1)	31(69)	9(20)	572

^a^ The median(IQR) percent error comparing predictions to observations.

^b^ The percentage of predictions that are over- or under-estimated compared to observations.

^c^ The percentage of predictions that are over- or under-estimated by at least 25% compared to observations.

^d^ The number of data points (not the number of subjects).

**Table 5:** Effect of lung capacity on predictions of ${\dot{V}}_{E}$ using measured FVC to calculate ${\dot{V}}_{E}$ according to Model D2. Model performance is shown for subjects with FVC measurements stratified into low, high, and normal FVC groups.

	percent error median(IQR)^a^	over (under)^b^	25% over (25% under)^c^	N^d^
subjects with measured FVC 85–115% predicted	-4.63(29.6)	41(59)	15(14)	2288
subjects with measured FVC < 85% predicted	0.422(22.7)	52(48)	6(6)	596
subjects with measured FVC > 115% predicted	2.57(27.1)	58(42)	15(10)	572

^a^ The median(IQR) percent error comparing predictions to observations.

^b^ The percentage of predictions that are over- or under-estimated compared to observations.

^c^ The percentage of predictions that are over- or under-estimated by at least 25% compared to observations.

^d^ The number of data points (not the number of subjects).

### Effect of FEV_1_

In obstructive airway disease such as asthma, bronchial constrictions can reduce expiratory flow during rapid exhalation without altering vital capacity [26]. The resulting reduced FEV_1_/FVC ratio is a classic trait of obstructive airway disease, and it is plausible that in such cases, the baseline FEV_1_ value would have a stronger influence on ${\dot{V}}_{E}$ than FVC. We therefore explored the influence of baseline FEV_1_ measurements on ${\dot{V}}_{E}$ predictions, but did not observe any improvement in model performance. It should be noted that since predictions of FEV_1_ assume no airway disease, there is no meaningful difference between models developed using predicted FVC and predicted FEV_1_ (though the parameter estimates for each are of course different). The performance of models using measured FEV_1_ as a predictor was essentially no different than models using measured FVC. However, this finding may be due to the fact that all subjects with lung function measurements had a FEV_1_/FVC ratio close to normal (median = 0.81), and we cannot draw a conclusion on the relative merits of baseline FVC versus FEV_1_ for purposes of estimating ${\dot{V}}_{E}$.

### Effect of age

For models including age as a predictor, but not height, sex, or FVC, the parameter estimate(standard error) for age is 0.45(0.023) when f_B_ is included and 0.43(0.024) when it is not. This implies that all else being equal, ${\dot{V}}_{E}$ would increase with age. However, previous studies have suggested that in adulthood, resting ${\dot{V}}_{E}$ is not sensitive to age independent of other factors [27]. In order to explore this discrepancy, we divided the dataset into two strata by age, successively using the ages 15–30 years as the cutpoint. In each case, the younger strata had a larger parameter estimate for age, and we found a negligible effect of age on ${\dot{V}}_{E}$ in strata consisting of persons over 24 years. Other factors that influence ${\dot{V}}_{E}$ during activity are known to be affected by age, including VO_2_ max, maximum voluntary ventilation, response to hypoxia, and FVC. [27] These factors lead to age-related differences in HR and f_B_ for the same level of activity, and as a consequence, models including HR, f_B_, FVC as well as age are equally predictive of ${\dot{V}}_{E}$ in the adult population as in the child or adolescent population. We did not observe an improvement in predictive performance by stratifying the model by age (regardless of the cutpoint), perhaps due to the reduction in statistical power resulting from stratification.

### Effect of height

Previous studies have found that HR is higher [28] and FVC is lower [22, 23] in persons of shorter stature. If HR and FVC are included as predictors of ${\dot{V}}_{E}$, the addition of height does not improve predictive performance and the parameter estimate is non-significant, suggesting that much of the effect of height on ${\dot{V}}_{E}$ is a result of the height-related changes to HR and FVC. If FVC is not included as a predictor however, the effect of height is pronounced and statistically significant. When comparing models including FVC but not height to models including height but not FVC, the predictive performance of the FVC models is better, particularly when using measured rather than estimated FVC. Taken together, these findings suggest that height does not have a large effect on ${\dot{V}}_{E}$ independent of its effect on lung capacity.

### Effect of sex

We evaluated the effect of sex in three different ways: including sex as a predictor, stratifying by sex, and cross-validating by sex (i.e. using males as the training set and females as the validation set, then vice versa). All three methods suggested a small but significant effect of sex on predictions of ${\dot{V}}_{E}$. Using sex as a predictor produced a statistically-significant parameter estimate for sex which implied that all else being equal (including FVC), ${\dot{V}}_{E}$ is 13% lower in females than in males. When stratifying by sex, the parameter estimates for HR and age were similar across strata while those for f_B_ and FVC were markedly different. In addition, cross-validation by sex was substantially worse than the random 10-fold cross validation. Taken together, these results suggest an effect of sex on ${\dot{V}}_{E}$ that is independent of FVC. Including sex as a predictor resulted in better-performing models than stratifying by sex, perhaps due to the reduction in statistical power resulting from stratification.

### Effect of race or ethnicity

Previous studies that have examined lung function in diverse populations have observed important differences associated with race or national origin. [22, 23] This diversity is likely the result of both differences in developmental environment and genetic factors [29, 30] such as adaptation to high altitude [31, 32]. We attempted to evaluate the effect of race or ethnicity in a similar fashion as the effect of sex. However, the compiled dataset was disproportionately composed of white subjects, and there were insufficient numbers of all other race or ethnicity categories to meaningfully evaluate each on its own. We instead used white and non-white race categories where non-white consisted of the African-American, Asian, and Hispanic categories. We acknowledge that this is not an ideal approach for assessing the role of human genetic diversity on ${\dot{V}}_{E}$. In particular, we note that there is likely a great deal of diversity within each race category, that each of the non-white categories are likely to be quite different from each other and that the Hispanic category does not necessarily identify genetic background and could include persons with various contributions of European, African, and Native American genetics. Stratifying Models D1 and D2 by race resulted in parameter estimates that were somewhat different from each other; but when cross-validating by race, the percent error was unchanged from random 10-fold cross-validation. It is conceivable that there is no effect of race independent of an effect of race on FVC; however, it is also conceivable that there is an effect of race but that this pooled data set was not sufficiently powered in non-white racial categories to detect that effect.

### Effect of lagged HR

HR and f_B_ have different response and relaxation times following stimuli [21], and the relationship between HR and ${\dot{V}}_{E}$ may be different when HR is increasing during activity than when it is decreasing. To parameterize this phenomenon, we evaluated models including either lagged or factorial terms for HR and f_B_ as predictors. Note that these terms are unavailable for the first several minutes of each participant’s session (for however many minutes are lagged or included in the factorial term), and this results in some loss of statistical power. Factorial terms performed better than lagged terms in all cases. The parameter estimates for HR factorials were significant (p < 1e10^-6^), but f_B_ factorials were not. Nonetheless, inclusion of these terms as predictors did not improve model performance, and we did not include them in our recommended models. It is possible that the direction of the HR trend (increasing or decreasing) is unimportant for predicting ${\dot{V}}_{E}$; however, this data set was primarily assembled from exercise tests of increasing intensity such that the vast majority of data points are from an increasing HR trend. It is therefore also conceivable that including factorial terms to identify the HR trend may be useful in predicting ${\dot{V}}_{E}$ in the post-maximum exertion time period, but that effect is not detectable with this dataset.

### Effect of “study”

We evaluated the possible systematic effects of which participating study collected data in two ways: we included a random effect for “study”, and we cross-validated by study (i.e., we in turn used data from each study as a validation set and data from the other seven studies as the training set). The random effect for study was very small in comparison to the random effect for subject, and the cross-validation results by study were not meaningfully different than the random 10-fold cross-validation. These results are shown in Table B and Figure D of S1 Text. Taken together, this suggests that were not large systematic differences in relationships between variables in data collected from the various contributing studies, and we therefore did not include a random effect for “study” in the final analysis.

### Comparison with previous studies

Several different models for predicting ${\dot{V}}_{E}$ have been previously proposed. The underlying methodologies for these models are diverse and include static estimates of ${\dot{V}}_{E}$ based on the type of activity, models based on energy expenditure, metabolic equivalents, oxygen consumption, HR, f_B_, or a combination of HR and f_B_. Most of these previously published models have not been cross-validated in a large sample. Dons et al. [13] recently compared the calculated ${\dot{V}}_{E}$ and air pollution dose using 16 different models on subjects using wearable sensors and is a co-author on this paper. This study found a very wide range of predicted ${\dot{V}}_{E}$. For some activities, the predictions differed by a factor of 2–4 using the same data as input. The application of previously-published models to our assembled dataset is shown in Table 6 along with the results of Models D1 and D2 from this paper. In addition to the random 10-fold cross-validation results, we have also included the results of cross-validation by study in this table as this may be a more fitting comparison for models from other studies. Please note that Table 6 only displays models that can be evaluated using data included in our dataset. Both Models D1 and D2 presented here have a substantially lower percent error than any previously published model. The best performing model evaluated by Dons et al. was that of Zuurbier et al. [6] When evaluated using our pooled data set, the performance of this model is substantially worse than either Model D1 or Model D2 with a median(IQR) percent error of 4.20(68.3)% as compared to -0.664(45.4)% and 1.20(37.9)% for Models D1 and D2 respectively.

**Table 6:** The results of applying previously-published ${\dot{V}}_{E}$ predictive models to this assembled dataset. For reference, the results of Models D1 and D2 from this paper are shown, including both random 10-fold cross-validation and cross-validation by study.

Model	percent error median(IQR)	Model parameters
Zuurbier et al. [6]	4.20(68.3)	males: ${\dot{V}}_{E}=e^{1.03+0.021HR}$
		females: ${\dot{V}}_{E}=e^{0.57+0.023HR}$
Ramos et al. [9]	12.0(67.2)	males: ${\dot{V}}_{E}=e^{1.17+0.02HR}$
		females: ${\dot{V}}_{E}=e^{0.99+0.02HR}$
Cozza et al. [8]	16.2(69.7)	${\dot{V}}_{E}=e^{0.58+0.025HR}$
Do Vale et al. [33]	-30.0(40.9)	${\dot{V}}_{E}={0.00071HR}^{2.17}$
McArdle et al. [34]	63.0(123)	${\dot{V}}_{E}=f_{B}(1.8028\cdot \ln\left(f_{B}\right)-3.8881)$
Greenwald et al. [14]	24.9(65.5)	${\dot{V}}_{E}={-4.247+0.0595HR+0.226f}_{B}$
model 1 (10-fold cross-validation)	-0.664(45.4)	see Table 3
model 1 (cross-validation by study)	-2.04(46.3)	see Table 3
model 2 (10-fold cross-validation)	1.20(37.9)	see Table 3
model 2 (cross-validation by study)	1.07(39.5)	see Table 3

### Limitations

Parameter estimates for all models in this study were calculated using predicted rather than measured FVC. By definition, these predictions are accurate for persons with average lung function, but this obscures the fact that there is a wide range of diversity in lung function values even for healthy individuals. The standard deviation for FVC predictions from the GLI study is approximately ±10% of the predicted value, and the lower limit of normal is approximately 20% lower than the predicted value. In addition, many persons do not have normal lung function, including people who are susceptible to the health effects of air pollution exposure. This includes asthmatics [35–37] and persons with chronic obstructive pulmonary disease [38]. Asthmatics frequently have lower lung function than non-asthmatics depending on phenotype and age of onset [39]. Furthermore, air pollution exposure itself is associated with decreased lung function [35, 40–43]. Given the large number of nominally healthy subjects included in this data set, it is plausible that there were approximately equal numbers of participants with FVC above and below the predicted value and that the parameter estimates are not biased. 82 of the 471 subjects included in the data set had an FVC measurement, and of these, fourteen had measured FVC more than 15% lower than the predicted value, and eight were more than 15% higher. As previously discussed, this small difference in lung function had an observable effect on ${\dot{V}}_{E}$ predictions, and this error was ameliorated by estimating ${\dot{V}}_{E}$ using the measured value of FVC instead. It is additionally possible that if the data set had included large numbers of participants with asthma or other airway disease or who otherwise had measured lung function substantially different than predicted, the calculated parameter estimates for Models D1 and D2 would be meaningfully different than reported here, and it is further possible that measured FEV_1_ would be a better predictor of ${\dot{V}}_{E}$ than measured FVC.

Another limitation of this paper was that all changes in ${\dot{V}}_{E}$ were driven by physical activity. It has been previously established that noise and anxiety affect ${\dot{V}}_{E}$ [44–47], and it is plausible that changes in ${\dot{V}}_{E}$ driven by noise or anxiety will have a different relationship with HR and f_B_ than those driven by physical activity. In the context of air pollution exposure, this would be relevant for persons in a loud or stressful transportation environment with elevated air pollutant concentrations. Additional research is necessary to determine if this is true, and if so, to what extent, and what parameters might be useful for accurately estimating ${\dot{V}}_{E}$ in persons experiencing noise, stress, or anxiety.

## Conclusion

We describe a method for estimating ${\dot{V}}_{E}$ in healthy individuals using HR as the continuously-measured predictor. Model accuracy and precision is improved by including continuously-measured f_B_ data as well. These predictions have been validated in a large diverse dataset comprised of 471 unique persons aged 4–80 years collected as part of eight independent studies. We found FVC to be an important factor in predicting ${\dot{V}}_{E}$; predicted FVC calculated according to a large well-powered study such as the GLI is a substantial improvement over not accounting for FVC; however, using measurements of FVC to estimate ${\dot{V}}_{E}$ further improved predictions, especially in persons with lung function higher or lower than normal. We additionally found age and sex to be important predictors; however, we did not find height or race to be important predictors independent of their influence on FVC. These models have been validated in individuals whose ${\dot{V}}_{E}$ is modulated in response to physical activity, and model results may not be accurate for predicting ${\dot{V}}_{E}$ that is modulated by stress, noise or anxiety. This method is more accurate and precise than other predictive models for estimating ${\dot{V}}_{E}$ and has the advantage of relying on predictors that are easily-measured in the field without specialized equipment.

## Supporting information

S1 Text Supporting information file. This file contains a text description and results of preliminary exploratory models. It includes three figures showing cross-validation results of these models as well as a figure showing the results of cross-validation by study of the Model D2 from the main text. Finally it includes a table for the random effects for subject, study, HR, and f_B_.
(DOCX)

S1 Data The deidentified pooled data file used for all models is included in a supplementary file labeled S1 Data. (TXT)
