# Supplementary material for: Estimating minute ventilation and air pollution inhaled dose using heart rate, breath frequency, age, sex and forced vital capacity: A pooled-data analysis
Source: PLoS One. 2019 Jul 9;14(7):e0218673. doi: 10.1371/journal.pone.0218673 (PMC6615621; doi:10.1371/journal.pone.0218673)
Supplement: S1 Text — This file contains a text description and results of preliminary exploratory models. It includes three figures showing cross-validation results of these models as well as a figure showing the results of cross-validation by study of the Model D2 from the main text. Finally it includes a table for the random effects for subject, study, HR, and fB. (DOCX) [file pone.0218673.s001.docx]

**Supporting Information File**

**Estimating Minute Ventilation and Air Pollution Inhaled Dose Using Heart Rate, Breath Frequency, Age, Sex and Forced Vital Capacity: a Pooled-Data Analysis**

Roby Greenwald

Matthew J. Hayat

Evi Dons

Luisa Giles

Rodrigo Villar

Djordje G. Jakovljevic

Nicholas Good

*Results from Modeling Approaches A, B, and C.* We explored numerous modeling approaches in order to identify the most appropriate and best performing predictive models, and we have labeled these approaches as follows: Approach A uses V̇_E_ normalized by forced vital capacity (FVC) as the dependent variable, Approach B uses V̇_E_ as the dependent variable and including determinant factors of FVC as predictors, Approach C the same as Approach B but also includes FVC as a predictor of V̇_E_, and Approach D uses log-transformed V̇_E_ as the dependent variable and log-transformed HR, f_B_, FVC, and subject-specific traits as predictor variables. As we describe in the main text, we ultimately selected Approach D models because they have better predictive performance and ease of use. For the sake of transparency, we present here the results of the best-performing models using Approaches A, B, and C. Parameter estimates are shown in Table A, and cross-validation results are shown in Figures A, B, and C. Approaches A, B, and C performed poorly for subjects at rest and occasionally produced negative predictions of V̇_E_ for subjects with HR of less than about 60 beats per minute. The minimum observed V̇_E_ for a subject at rest was 0.78·FVC, and we therefore substituted 0.78·FVC for any predicted ventilation value less than that for models using Approaches A, B, or C. To provide a reference, the comparable model using Approach D has a median(IQR) percent error of 1.20(37.9)%, nearly identical to the best Approach A model, but without having to adjust for subjects at rest.

*Effect of “study”.* We examined the possible effects of systematic differences in data collection methodologies between the contributing studies in two ways. First, we included a random effect for “study”, a categorical variable from 1-8 that corresponds to which study collected the data. Second, we cross-validated by study such that the data from seven studies comprised the training set and the data from the eighth study comprised the validation set. This was repeated until all eight studies had served as the validation set. The random effects results for Model D2 are shown in Table B. The variance component estimate of the random effect for “study” is 0.0071, a negligible value in comparison to that for subject. The results of cross-validation by study are shown in Figure D. These results show little difference with the random 10-fold cross-validation shown in Figure 2 of the main text. Taken together, these findings suggest there were not systematic differences or biases in data collected from the various contributing studies, and we therefore did not include a random effect for “study” in the final analysis.

| **Table A**. Parameter estimates and cross-validation results of general linear mixed models using modeling Approaches A, B, and C. For parameter estimates, the first row is the estimate(95% confidence intervals), and the second row is the p-value. Percent error refers to the difference between predictions and observations from cross validation, and values are median(IQR). HR is in beats per minute, f_B_ is in breaths per minute, age is in years, height is in centimeters, sex is 0 for males and 1 for females, and FVC is the GLI predicted value in liters. In all cases, the estimated value of V̇_E_ is the maximum of either the value calculated from the parameter estimates or 0.78·FVC. | | | |
| --- | --- | --- | --- |
|  | Approach A | Approach B | Approach C |
| dependent variable | V̇_E_/FVC | V̇_E_ | V̇_E_ |
| intercept | 182(174,191)  p < 10^-6^ | -2240(-3990, -493)  p = 0.015 | -1796(-3560, -33.5)  p = 0.0465 |
| ln(HR) | -60.2(-63.3, 57.1)  p < 10^-6^ | 1640(899, 2380)  p = 6.6·10^-5^ | 1520(778, 2270)  p = 7.48·10^-5^ |
| ln(HR)*ln(HR) | 4.90(4.48, 5.33)  p < 10^-6^ | -262(-341, -183)  p < 10^-6^ | -251(-330, -172)  p < 10^-6^ |
| ln(f_B_) | -27.7(-29.5, -26.0)  p < 10^-6^ | -127(-135, -119)  p < 10^-6^ | -127(-135, -119)  p < 10^-6^ |
| ln(HR)*ln(f_B_) | 6.94(6.56, 7.31)  p < 10^-6^ | 31.1(29.4, 32.7)  p < 10^-6^ | 31.1(29.4, 32.7)  p < 10^-6^ |
| age | -0.400(-0.466, -0.335)  p < 10^-6^ | -0.803(-1.11, -0.501)  p < 10^-6^ | -0.750(-1.06, -0.446)  p = 1.94·10^-6^ |
| ln(height) | -.367(-4.80, -2.53)  p < 10^-6^ | 579(240, 918)  p = 0.00152 | 489(147, 832)  p = 0.00534 |
| ln(HR)*age | 0.0996(0.0848, 0.114)  p < 10^-6^ | 0.197(0.128, 0.266)  p < 10^-6^ | 0.196(0.127, 0.265)  p < 10^-6^ |
| ln(HR*height) | - | -372(-516, -228)  p = 5.3·10^-6^ | -349(-493, -204)  p = 3.16·10^-6^ |
| ln(HR^2^*height) |  | 5540(4010,7070)  p < 10^-6^ | 5330(3790, 6860)  p < 10^-6^ |
| sex | -0.378(-0.630, -0.126)  p = 0.0035 | -3.06(-4.07, -2.05)  p < 10^-6^ | -1.74(-2.91, -0.561)  p = 0.00397 |
| FVC | - | - | 3.01(1.84, 4.18)  p < 10^-6^ |
| percent error | -0.860(37.8) | -0.942(40.7) | 0.746(39.5) |

| **Table B**. Random effects of Model D2. | | | |
| --- | --- | --- | --- |
| Groups | Name | Variance | Standard Deviation |
| subject | intercept | 3.75 | 1.94 |
|  | HR | 0.247 | 0.497 |
|  | f_B_ | 0.180 | 0.424 |
| study | intercept | 0.00707 | 0.0841 |
| residual |  | 0.0255 | 0.160 |


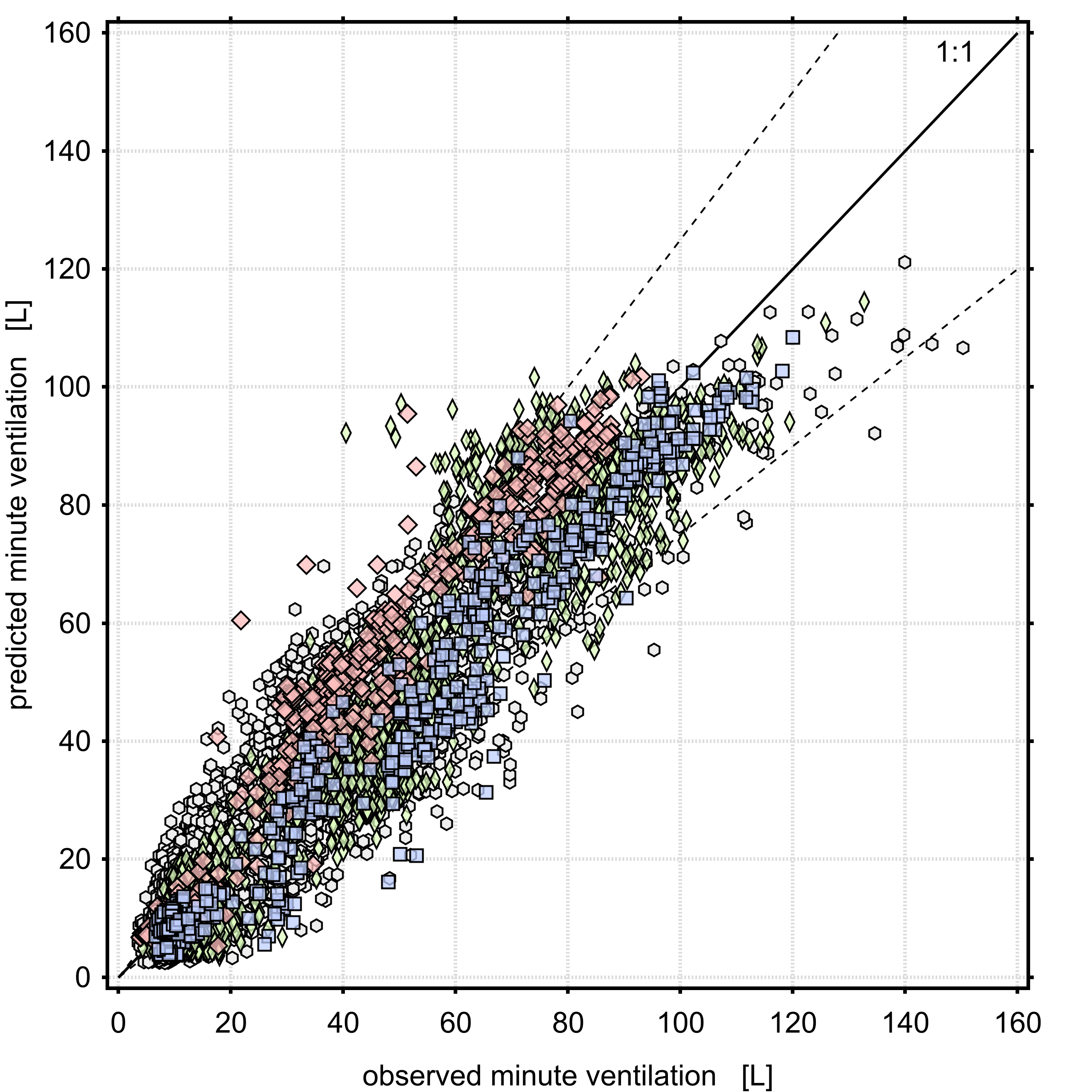


Figure A. Cross validation results for the best Approach A model. Parameter estimates for this model are provided in Table A. The median(IQR) percent error from cross-validation for this model is -0.860(37.8)%. Gray hexagons are persons without an FVC measurement; green diamonds are persons with measured FVC = 85-115% of the predicted value; pink diamonds are persons with measured FVC < 85% predicted, and blue squares are persons with measured FVC > 115% predicted. Dashed lines are ±25% error.


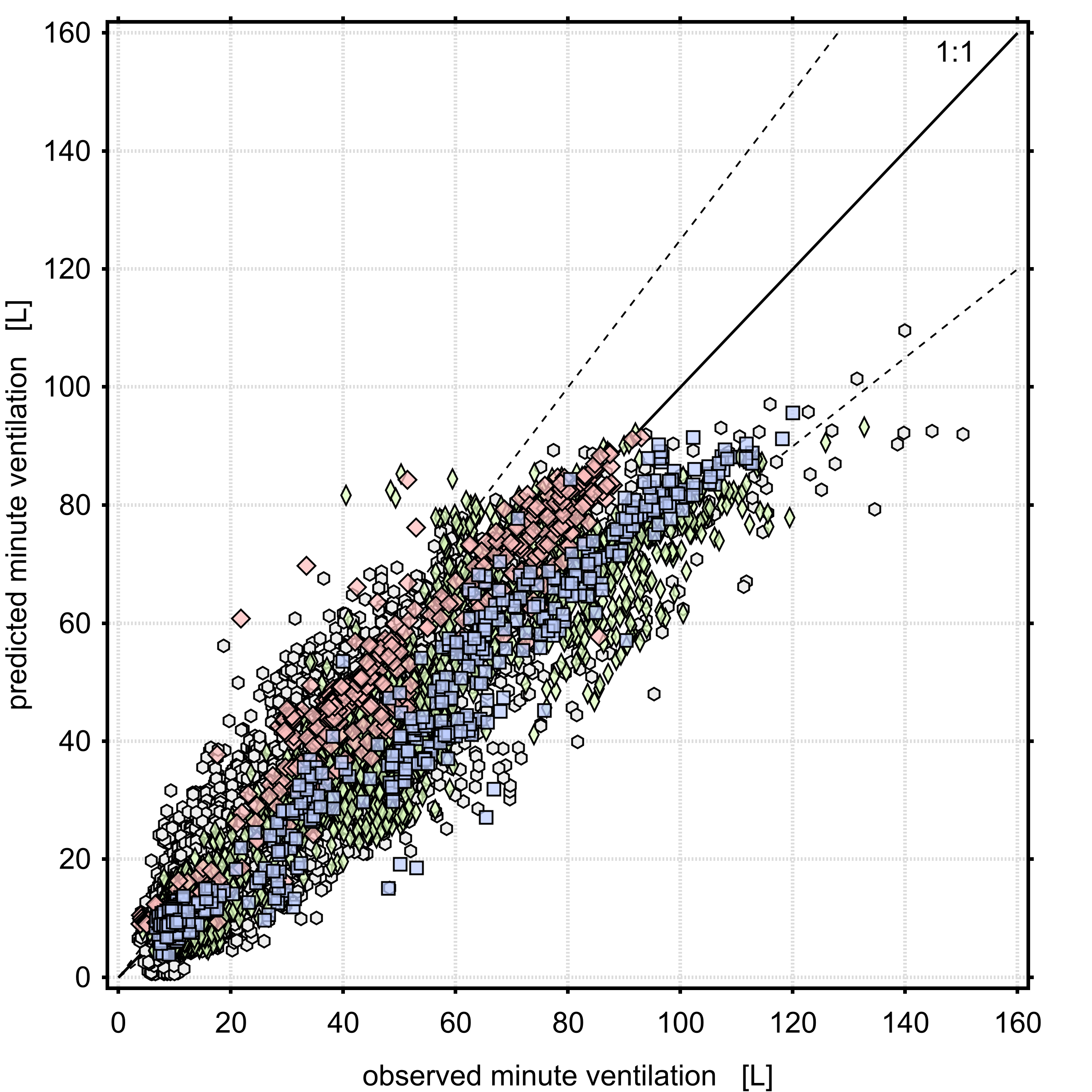


Figure B. Cross validation results for the best Approach B model. Parameter estimates for this model are provided in Table A. The median(IQR) percent error from cross-validation for this model is -0.942(40.7)%. Gray hexagons are persons without an FVC measurement; green diamonds are persons with measured FVC = 85-115% of the predicted value; pink diamonds are persons with measured FVC < 85% predicted, and blue squares are persons with measured FVC > 115% predicted. Dashed lines are ±25% error.


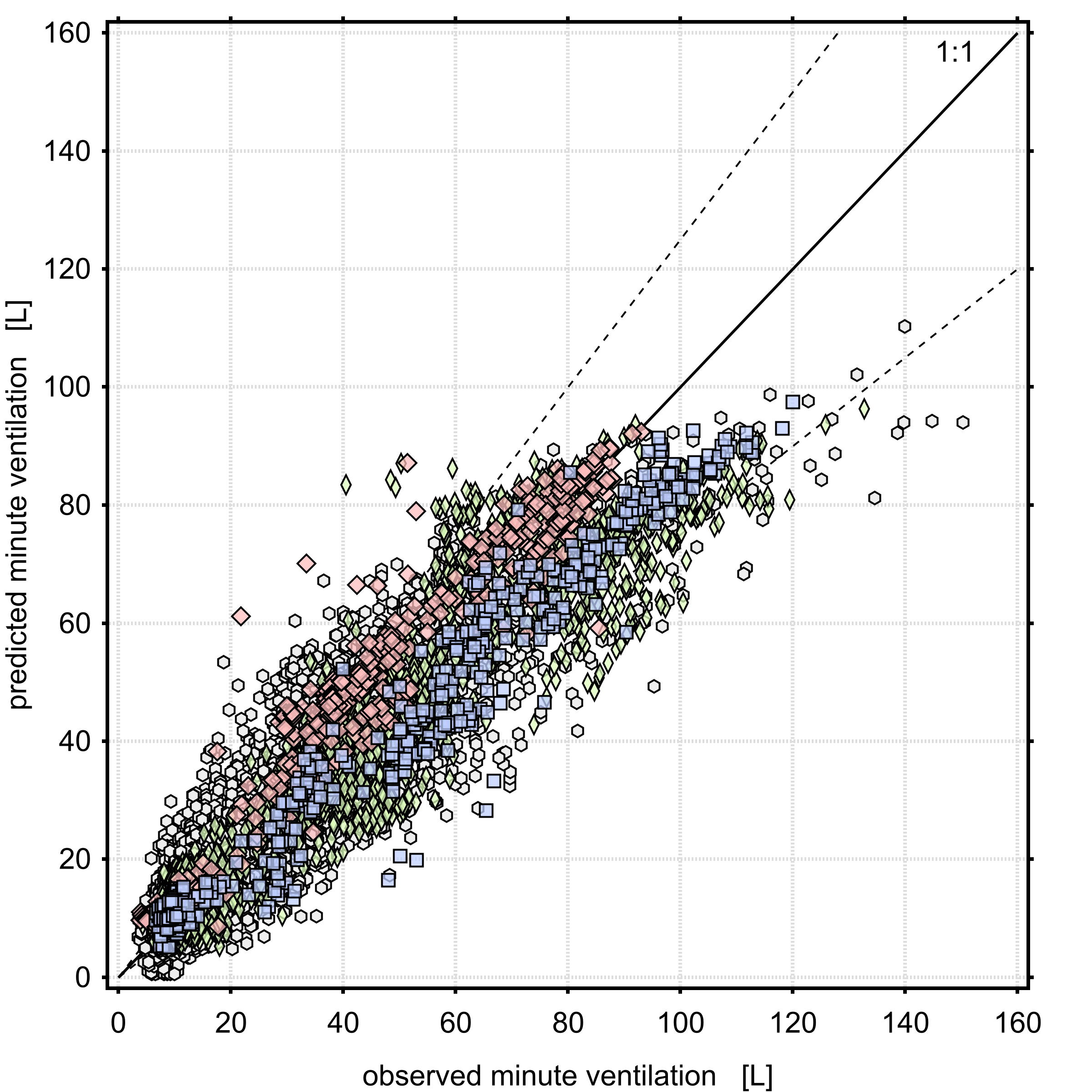


Figure C. Cross validation results for the best Approach C model. Parameter estimates for this model are provided in Table A. The median(IQR) percent error from cross-validation for this model is 0.746(39.5)%. Gray hexagons are persons without an FVC measurement; green diamonds are persons with measured FVC = 85-115% of the predicted value; pink diamonds are persons with measured FVC < 85% predicted, and blue squares are persons with measured FVC > 115% predicted. Dashed lines are ±25% error.


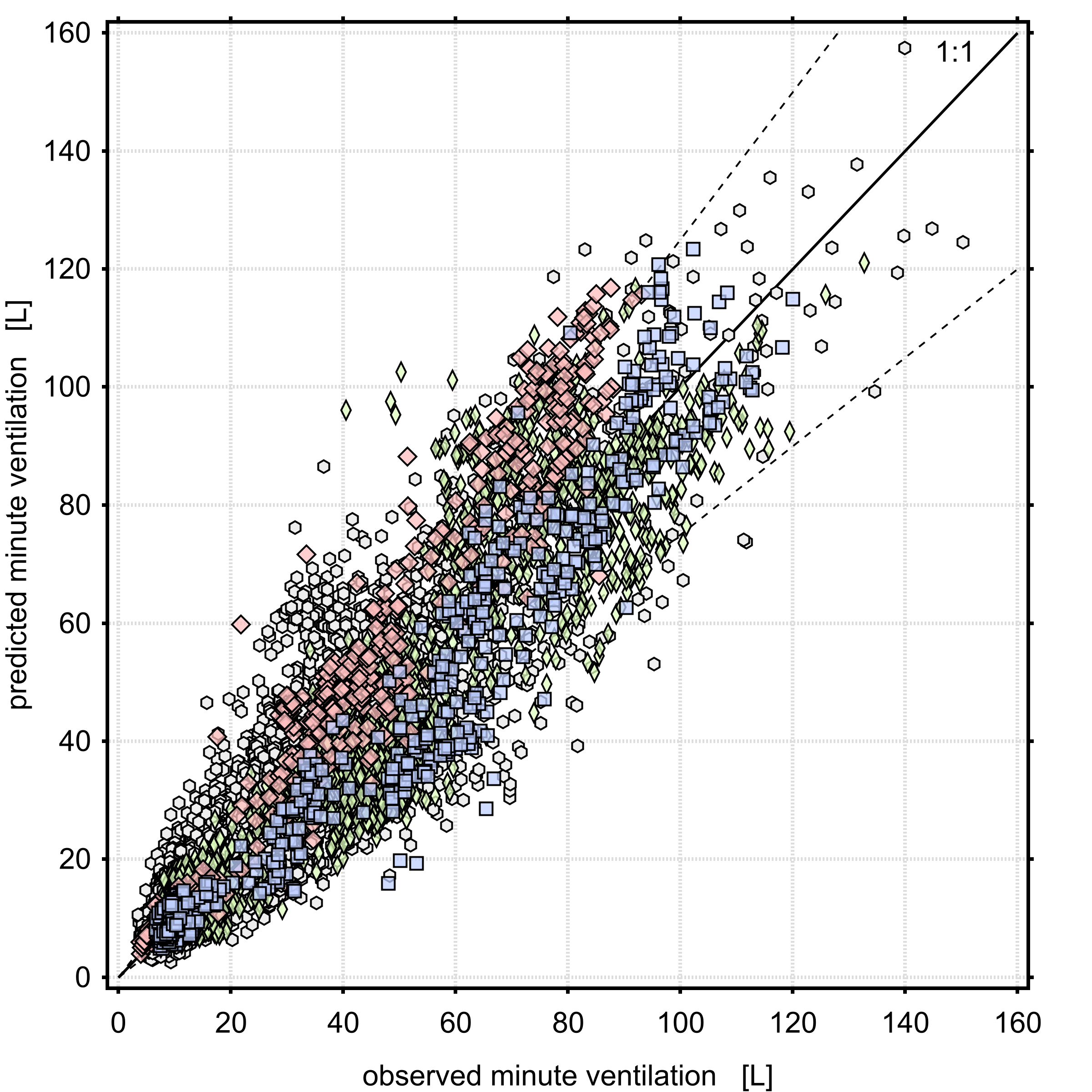


Figure D. Results of cross-validation by “study” for Model D2. The median(IQR) percent error from this cross-validation is 1.07(39.5)%. Gray hexagons are persons without an FVC measurement; green diamonds are persons with measured FVC = 85-115% of the predicted value; pink diamonds are persons with measured FVC < 85% predicted, and blue squares are persons with measured FVC > 115% predicted. Dashed lines are ±25% error.
